# Implementing artificial intelligence education for middle school technology education in Republic of Korea

**DOI:** 10.1007/s10798-023-09812-2

**Published:** 2023-02-20

**Authors:** Woongbin Park, Hyuksoo Kwon

**Affiliations:** 1 Gajaeul Middle School, Seoul, Republic of Korea; 2 Department of Technology & Home-Economics Education, Kongju National University, #102 Human Ecology Building, Gongjudaehak-Ro 56, Gongju-si, Chungcheongnam-do 32588 Republic of Korea

**Keywords:** Artificial intelligence education, Free semester, Technology education, AI competency, Attitudes toward technology

## Abstract

The purpose of this study is multifold: First, to develop an educational program using artificial intelligence (AI) in middle school free semester system of South Korea. Second, to verify the program’s effectiveness, the study clarified the definition of AI and AI education and considered their meaning in technology education. This study used three steps: preparation, development, and improvement. In the preparation step, this study set the theme and purpose of the AI program and selected the free semester activity type “theme selection activity.” After analyzing the technology curriculum and extracting AI-related elements in the development step, this study laid out the program for 16 h of class time. In the improvement step, to augment the program’s validity, the researcher revised and supplemented it as a whole through expert consultation. This research differentiated the developed program from the AI education program of other subjects and specialized it, focusing on the specificity of technology education. The study emphasized the social impact of the latest technology, ethics of AI, physical computing using AI, and technological problem-solving activity using AI. The final developed program was applied to the students, and students participated in a pretest and posttest. The study used the PATT and AI competency test tools. The PATT results showed a significant increase in the mean of both constructs in “interest in technology” and “career aspirations in technology.” In AI competency, the mean of two constructs increased significantly in “social impact of AI” and “AI performance.” In particular, “AI performance” showed the largest increase. There was no statistically significant change in “interaction with AI.” The study results confirmed that the developed AI program was effective in technology education and career exploration, which is the primary purpose of the free semester. In addition, it was possible to confirm the technology educational value of the AI education program centered on technological problem-solving. These research results have implications for bringing AI into technology education.

## Introduction

As the field of education emphasizes career education, each provincial education office tries to develop students’ dreams and talents through various programs and teaching–learning methods in Republic of Korea. Career experience is implemented from the elementary school level while high schools aim to deepen career education. Meanwhile, in middle schools, the Ministry of Education implemented a nationwide free semester and free year system (from now on “free semester”) in 2016 (Ministry of Education, 2015a). As a result, quantitative grading was removed, leaving only qualitative evaluations in the free semester system. Freed from paper-based evaluations, students can explore career paths of their dreams and talents through various programs (Kim & Hong, 2021).

On the other hand, despite good intentions, there are critical differences of opinion between the Ministry of Education and the Office of Education, teachers in the field and students. Developing curriculum for free semester classes involves significant research and it may be challenging to conduct fair evaluations (Shin et al., 2015). Thus, teachers struggle to provide quality classes to students, and students perceive the free semester system as a “play semester,” and do not concentrate on classes. As a result, difficult situations for teachers and students often arise in class operations, which also cause parental dissatisfaction (Shin et al., 2018).

Meanwhile, although technology education is a subject with many areas that one can deal with according to each one’s characteristics, research on theme selection activity and career education in the free semester is insufficient. Therefore, in-service technology teachers are reluctant to take on these classes because of the additional workload they imply. In addition, there are problems such as a lack of practical materials, lack of practice space, and lack of programs (Kim, 2017). Meanwhile, apart from career education, the area that the current government (joint policy of each related ministry) is most actively promoting is artificial intelligence (AI)-related industry (Government, 2019). The Ministry of Education (2020) created various AI and associated subjects in the 2022 revised curriculum for application starting in 2025, including “Programming,” “AI Basic Principles,” “AI Utilization,” “AI Ethics.” Metropolitan and provincial offices of education are already implementing AI-related projects. In addition, schools are applying AI-related education, such as operating AI education leading schools, and constructing AI laboratories (Busan Metropolitan City Office of Education, 2021; Seoul Metropolitan Office of Education, 2021).

In the results of a study on Korean teachers' perceptions related to AI education, the demand for using AI in real life and learning situation was high, but the demand for learning the concept and principle of AI were relatively low (Kim & Han, 2020). Additionally, teachers recognized the necessity of integrating AI to the problem-solving process with other subjects (Kim & Han, 2020). This finding shows that teachers recognize “real-life problem-solving and inter-subject convergence education through AI” as the direction of AI education. On the other hand, the study found low teacher awareness of the need for education in specialized fields such as “the concept and principle of AI” and “AI development.”

However, research on systematically applying AI and machine learning in education is insufficient (Tedre et al., 2021). In addition, Lee’s (2021) questionnaire-based study showed a very low level of confidence among in-service teachers, with 65.4% of elementary school teachers in charge of AI education answering, “I do not know how to apply AI convergence education in the subject.” It is not expected that this pattern will be significantly different in middle school.

Despite the high demand for AI, there is not enough education or support for field teachers or preservice teachers, creating a gap between the policy and field atmosphere. Even though the Ministry of Education and related ministries actively promote free semester and AI education projects, there are still many difficulties in applying them in school. Moreover, research in AI and software-related fields or free semester research in technology education is insufficient (Cho, 2017; Lee, 2018). With the above background, this study developed an AI program that can be applied immediately by technology teachers teaching the free semester, measuring its effectiveness after application with students.

### Purpose of research

This study developed a program to bring AI into the technology education free semester in middle school. In addition, the study measured the effectiveness of AI education programs in technology education, attitudes toward technology, and AI-related competencies. The two objectives of the study are:

First, develop an artificial intelligence theme selection activity for application in technology education during the free semester.

Second, analyze the effect of the artificial intelligence theme selection activity on students’ attitudes toward technology and AI competencies.

## Background

### Free semester in Korean middle school

The free semester is an educational policy introduced to improve public education in South Korea. Similar examples abroad include the “transition year programme” in Ireland, “prao” in Sweden, “afterschool” in Denmark, and “gap year” in the United Kingdom (Kim & Choi, 2014). These educational policies reduce the pressure on learners to evaluate and provide experience-centered classes. As a result, students can explore their career paths and enable self-development according to their aptitudes. Furthermore, at the national level, governments applied free semesters to improve public education and to increases the reliability of school education.

The Ministry of Education (2015a) established the “Middle School Free Semester Implementation Plan” in 2015, and implemented the free semester in all middle schools in 2016. The program expanded the domestic free semester from one-semester “free semester” in 2016 to a one-year “free year system” in 2018. Currently, the government is promoting it to link the free year system with the general semester and gradually expand the program (Ministry of Education, 2017; Park & Hong, 2021). The government and many studies use the terms free semester and free year system interchangeably (in this study, unified as “free semester”). Students complete a flexible curriculum during the free semester, allowing them to participate in student-participatory classes and various career experience activities to discover their dreams and talents.

The Ministry of Education (2015a) presents the purpose of the free semester as “finding dreams and talents by exploring aptitudes and future,” “transitioning to future core competency-enhancing education,” and “happiness education that satisfies all students, parents, and teachers.” The activities of the free semester in South Korea are composed of four types as shown in Table 1. Generally, “career exploration activity” and “club activity” have been implemented for career guidance teachers. In practice, middle school teachers take “theme selection activity” and “art-physical activity.” Moreover, “art-physical activity” occurs in arts and physical education subjects.

**Table 1:** Organization and operation of ‘free semester activities’

Free semester activities	Purpose and example
Career exploration activity	Purpose: to conduct systematic career education so that students can design their own future by exploring their aptitudes and talents Example: career examination, invitational lecture, portfolio creation activities, field experience activity, job tour, mock entrepreneurship activity, etc
Theme selection activity	Purpose:to motivate learning by operating systematic and professional programs tailored to students' interests and interest Example: drama and society, 3D printer, webtoon, happiness education, financial and economic education, smartphone app creation, etc
Art-physical activity	Purpose: to develop the talents and potential of students through diverse and substantial arts and physical education Examples: plays, musicals, orchestra, lyrics and composition, mural painting, design, soccer, basketball, sports league, etc
Club activity	Purpose: to develop potential abilities by autonomously choosing and performing activities based on common interests of students Example: literary discussion, line dance, science experiment, astronomical observation, photography, video, local art tour, etc

There are contrasting studies on the effectiveness of the free semester. Previous studies related to the career effect, which is one of the basic purposes of the free semester, confirmed various effects such as career decision self-efficacy, career competency, and self-efficacy (Hwang, 2019; Lee et al., 2016). Meanwhile, one longitudinal study of multiple middle schools showed that the free semester did not affect career maturity and self-efficacy (Lee, 2020). Another study confirmed “interest in science, technology, engineering, and mathematics” and “self-efficacy and career effects” of inquiry learning that applied convergence education in science free semester classes (Jeong & Lee, 2017). In addition, a qualitative study targeting secondary school teachers looked at the difficulties encountered in the science free semester classes (Kim & Choi, 2019). In the subject of mathematics, a study confirmed the change in “career exploration ability” and “attitude toward statistics” through the free semester statistics program (Kang et al., 2017). Additionally, another study looked at the link between convergence education research and engineering tools and 3D printers to mathematics education (Cho et al., 2014; Kang et al., 2016).

As mentioned above, the development of the free semester program for convergence education that deals with the elements of technology takes place in the subject, not the technology. In particular, Jeong and Lee’s (2017) study shows the practical possibility of free semester convergence education in technology education. In addition, 3D design and printing applied to mathematics education in the free semester system are tools frequently used in technology education, showing the possibility of realization in the free semester. However, unlike despite extensive research on technology education, there is currently a lack of research related to technology subject based free semester programs or evaluation. Accordingly, in-service technology teachers are experiencing difficulties in free semester classes (Kim, 2017; Kim & Lee, 2019; Lim & Lee, 2019; Um, 2016). Solving this problem requires empirical research on the free semester in technology education.

### Artificial intelligence in education

In 1950, British mathematician and electrical engineer Alan Turing proposed a test distinguishing between intelligent machines and humans. After his first in-depth review, a full-scale and thorough study of artificial intelligence began. Mathematician Marvin Minsky founded the Massachusetts Institute of Technology (MIT) Artificial Intelligence Lab and made significant contributions to the development of artificial intelligence. Among the latest artificial intelligence technologies, “perceptron (perception + neuron),” the core of neural network algorithms, was first mentioned by Frank Rosenblatt in 1958 (Rosenblatt, 1958). After that, Marvin Minsky and Seymour Papert published “Perceptrons,” and artificial intelligence technology developed further to today’s machine learning and deep learning (Minsky & Papert, 1969). Since 2000, there has been significant artificial intelligence development, with active research and application in broad industrial fields from management to manufacturing (Liu et al., 2018; Peres et al., 2020; Ruiz-Real et al., 2021).

### Artificial intelligence education

Seymour Papert, a mathematician and computer scientist who co-wrote “Perceptrons” with Marvin Minsky, greatly influenced AI education. Influenced by Jean Piaget, Seymour Papert significantly contributed to computer education and maker education and was interested in mathematics education using computers (Sung, 2018). Papert (1980) discusses his ideas about education in his book “Mindstorms: Children, Computers, and Powerful Ideas.” He said that learning spontaneously by interacting with the environment is the core of Piaget’s education theory and that computers make learning efficient. Therefore, he paid attention to computer-use education, simultaneously discussing AI education in his book. He defined AI education in terms of cognitive science, arguing for materializing metaphysical thinking in order to create intelligent machines (artificial intelligence). He expected students’ improved thinking processes through clarification of their thinking processes. For this reason, Seymour Papert argued the necessity of AI education.

Currently, there is a global effort to apply AI in the education field. Moreover, the field is actively discussing “what and how to teach students” in the era of AI. For instance, the Computer Science Teachers Association (CSTA) proposed “Computer Science For K-12 Standards” in the United States. Furthermore, the CSTA formed a joint council for AI education in 2018 with the Association for the Advancement of Artificial Intelligence (AAAI). After that, CSTA and AAAI created the Artificial Intelligence for K-12 Standards (AI4K12), proposing five big ideas for AI education, and are making efforts in AI education (Touretzky et al., 2019). K12-AI argues that students in the age of AI will have a fundamentally different relationship with technology than previous generation students. It also emphasizes teaching students in constructivist learning, design, and creative thinking to become citizens of the age of AI (Ali., 2019). In addition, in K12-AI, Wong stated that students should receive compulsory education to acquire AI literacy (manipulation and utilization of AI technology). They also argued that students need compulsory education to enter their respective professions as skilled workers. Finally, they emphasized the necessity of teacher and school system innovation and stakeholder cooperation (Wong., 2020).

Due to AI technology characteristics, the field also discusses educational perspectives on AI. For example, Luckin et al. (2016) said that Artificial Intelligence in Education (AIED) goes beyond educational technology and uses the sophistication of AI to increase learning efficiency. Furthermore, Holmes (2019) discusses AI education and classifies it into “Learning with AI” and “Learning about AI.” He also suggested dividing AI into “What we teach” and “How we teach it.” As such, AI-based education refers to an approach to AI in teaching and learning methods like the previous education using information and communication technology (ICT) (Busan Metropolitan City Office of Education, 2019).

Most see AI as a tool, but some educate purely on AI, approaching AI education as an extension of science, technology, engineering, and mathematics (STEM) education (Hong et al., 2020). The main concern is understanding AI and educating students about AI-related technology according to their level. For example, “5 big idea in AI” of AI4K12, which studied AI education standards, is representative. AI4K12 presents “perception, representation & reasoning, learning (machine learning, deep learning), natural interaction, and social impact” in AI as five core ideas (Touretzky et al., 2019). In addition, the topics of AI education involve AI ethics. Coeckelbergh (2019) pointed out the biases created by machine learning go beyond much-discussed privacy protection and discussed various ethical issues, such as the attribution of responsibility for these issues and the problem of unemployment, extending beyond the individual level to social issues. This point of view is similar to that of the Ministry of Education (2020). They added the subjects of “Artificial Intelligence Ethics” as well as “Artificial Intelligence Mathematics” and “Artificial Intelligence Fundamentals” to educate on AI itself. Meanwhile, AI ethics deals with the impact of AI technology on society, and there is an intersection with seeing AI as a tool of education.

In addition, educational researchers are actively conducting studies in AI-related fields, such as “computational thinking” and “SW competency,” reflecting competency-based evaluation (Choi, 2019). However, AI-related research has only recently emerged as a research topic, and investigation of the definition and evaluation of AI competency is insufficient (Min & Shim, 2021). Kim and Lee (2021) conducted a study to measure AI literacy for basic research related to students’ AI evaluation. Koh (2020) considered the mathematics curriculum to cultivate AI competency in mathematics education. A previous study related to AI evaluation developed a tool that can test the attitude toward AI to suit each middle and high school (Kim & Lee, 2020a, 2020b). Han (2020) developed an instrument to measure changes in attitude and efficacy toward AI in AI project classes. Another study developed and evaluated the fourth industrial revolution-related technology education program, including AI in technology education (Lee et al., 2019). Although there have been several studies related to AI evaluation, there have been few studies that actually dealt with AI education and evaluation in depth in Korea (Kim et al., 2021; Min & Shim, 2021).

As mentioned above, different subjects apply AI education in various ways. For example, in science education, a study intervention helped learners understand scientific principles through construction practice by building an AI-based mixed reality system (Yannier et al., 2020). Further, a study analyzed AI tools for application in computation and AI tutoring in mathematics education (Van-Vaerenbergh & Pérez-Suay, 2021). In addition, researchers frequently conduct studies using translation services, chatbots, and AI speakers in English education, according to the subject characteristics (Kaharuddin, 2021; Li et al., 2021; Wang & Petrina, 2013). Although not secondary education, a study of STEM-based AI education programs for non-engineering college students showed improvement in AI literacy (Lin., 2021). While research in AI education is primarily about either “learning with AI” or “learning about AI,” studies of science and mathematics education show that approaches with both characteristics are also possible.

Meanwhile, for trends in computer education-related research, Min and Shim (2021) analyzed related research topics in South Korea secondary education from 1998 to 2020 using topic modeling techniques. The results showed programming and SW were the keywords with the highest frequency from 2007 to 2018. Nevertheless, it showed that AI, which had never made it to the top 10 keywords until 2019, was the number one research topic. In fact, teachers” interest in AI is high in the current school field, and teachers are also aware of the need for AI education (Ryu & Han, 2018). On the other hand, despite the central position of AI and machine learning in the modern computing field, studies on systematically learning machine learning in education-related research are insufficient (Tedre et al., 2021). In fact, in a study examining the perception of elementary school teachers in charge of AI education, it was found that 65.4% of teachers lack self-confidence (Lee, 2021). Additionally, Shin (2020) showed that teachers negatively perceive the quality and evaluation of teaching–learning in education using AI. Although social and educational academics are very interested in AI research, the school is somewhat different from external expectations.

### Artificial intelligence and technology education

Studies on education methods using AI tools and AI education in STEM are underway. However, the number of studies in technology subjects is small. Software education and computational thinking related to AI also lack the number of studies in technology education compared to other subjects (Lee, 2018, 2019). Kim (2021) analyzed research trends related to AI in elementary and secondary education through topic modeling and showed that all keywords included “technology.” Still, there were very few studies on “technology education.” Lim (2020) said that technology education justifies AI education and that in the age of AI, technology education needs direction and active research. For example, in a study of elementary and secondary teachers, teachers answered that “AI education is using AI in life.” Furthermore, teachers answered that it is most important for students to integrate AI with other subjects or apply it to problem-solving processes (Kim & Han, 2020). In a cognitive analysis study of AI-using education, elementary school teachers said that “used to aid in class” is appropriate for AI in education (Han, 2020). In addition, educators recognized the “problem-centered learning approach” as the most appropriate (Han et al., 2020). The core goal of technology education is to solve technological and practical problems in the real world through a convergence approach (Custer, 1995). Thus, centering technology education is very effective for AI education.

A study by Lim (2020) suggested the direction in technology education and discussed the justification of AI education in technology curriculum. In addition, Kim’s (2020) study applied maker education methodology based on AI and physical computing in program development. Furthermore, Lee and Lee (2021) developed a program to select the core concepts of AI and apply them to technology education. In addition to AI, a study also selected the core interlocking technology of the fourth industrial revolution for application in middle school technology education, developed a program, and measured the effect (Lee et al., 2019). Although it is not direct, there have also been studies on robot education and developing bio-mimicking robot education programs in technology education (Kim, 2019; Kim & Jeong, 2006). Research on these AI-related technologies can serve as a reference for the direction of technology education.

As mentioned above, studies related to AI education can have many implications for technology education. For example, Papert (1980) expected that learners would improve their thinking in clarifying the thinking process to create AI. His research suggests that AI education can affect mathematical thinking and learners’ cognitive ability. In addition, although education can approach AI as a tool in computer science or educational methods, AI education from the STEM perspective can also affect technology education. Educators can teach AI as cutting-edge technology, and students can learn social changes in technology education. In addition to the technology aspect, the ethical problems of AI pointed out by Coeckelbergh (2019) are highly related to technology education’s ICT ethics. Moreover, research on AI conducted to realize each subject’s purpose in STEM-related subjects (science, mathematics) can indirectly provide directions for technology education.

## Method

### Research design

Based on the research purpose and literature review, this study developed an AI education program as a free semester theme selection activity in middle school technology education. The study applied the intervention as an actual free semester class in a middle school in Seoul. The program was implemented for eight weeks at two hours per week for 16 h. Most of the courses were face-to-face, but due to the COVID-19 virus, some courses (weeks 1 and 2) were real-time interactive video classes. The study applied the AI education program to 23 middle school students who participated in the experiment, conducting “technology attitude” and “AI competency” tests. The study used the experimental method of a single group pretest and posttest.

### Participants

The study implemented the program as a free-semester theme selection activity, allowing students to select it in the same way as the general free-semester program selection method. Although each school's situation is different, students generally have four theme selection programs to choose from depending on their career path, and this program was chosen as one of them by the participants. Before the program started, the researcher informed the students and parents of the study’s research ethics. The Institutional Review Board (task management number 2021–73) reviewed and approved this study. There were a total of 23 volunteer participants. The students were all first graders (12 ~ 13 years old) attending the middle school free year system, and there were 19 male students and 4 female students, for a total of 23 students. The students have experience in SW education in elementary school practical subjects. Students had a learning experience (2-h class) about the impact of AI on society with the subject of future technology and social change in the technology class in the first year of middle school. Educators introduced information subjects in the second year, so students did not receive education on related content.

### Instruments

This research conducted a pretest and posttest of a single group to investigate the study’s effect. The study performed quantitative tests through two instruments on “technology attitude” and “AI competency” before and after the program. After completing the program agreement, the researcher conducted a pretest before the program application and a posttest after the end of the program. The environment did not include the researcher (i.e., a teacher in charge); thus, students completed the test remotely at any time from one to three days after the last class.

The study piloted the test tool on 200 first-year middle school students to verify its reliability before application to study participants. As a result, the reliability of 19 items of The Pupils’ Attitude toward Technology (PATT) was 0.930. In the AI competency test that measured four constructs, the reliability of 12 items of three constructs was 0.812. Meanwhile, the reliability of one construct was lower than 0.7 (a characteristic of AI), so this study excluded it from test tool.

#### PATT (The Pupils’ attitude toward technology)

The PATT tool, developed by Bame et al. (1993), tests students’ attitudes toward technology. In South Korea, the study of Lee (2008) applied a five-point Likert scale after translation. This tool consists of six constructs, each with “interest in technology,” “perceived gender patterns in technology,” “perceived difficulties in the technology,” “consequences of technology,” “technology and curriculum,” and “career aspirations in technology.”

This study measured two constructs among the six constructs in the original instrument after the reviews of previous studies on PATT. This study measured “interest in technology” and “career aspirations in technology.” as shown in Table 2.

**Table 2:** The Pupils’ attitude toward technology

No	Items
1	When something new is discovered, I want to know more about it immediately
2	I would like to know more about computers
3	I want to watch YouTube about technology
4	There should be more TV and YouTube contents about technology
5	If there was a school club about technology, I would certainly join it
6	I think out-of-school experiential learning activities about technology are boring
7	I am not interested in technology
8	I enjoy repairing things at home
9	I think machines are fun
10	A technological hobby is fun
11	I will probably choose a job in technology
12	I will not consider a job in technology
13	I do not understand why anyone would want a job in technology
14	I would enjoy a job in technology
15	I would like a career in technology later on
16	Working in technology would be fun
17	Most jobs in technology are boring
18	Working in technology would be interesting
19	With a technological job your future is promised

Previous studies related to attitudes towards technology showed that middle school students’ interest in technology subjects was not high. The degree of interest differed depending on whether there was an experiment or practice (Kim, 2006). In addition, middle school students recognized the high importance of technology, but researchers found a relatively low perception of “interest in technology subjects” or “helpful in real life” (Lee, 2008). Therefore, Lee (2008) said that it is necessary to develop a program that can stimulate students’ intellectual curiosity or arouse interest, based on a survey on the perception of technology. Therefore, this study measured “interest in technology” after developing the program. This study reinterpreted old-fashioned questions such as “I want to read a technology-related magazine’ in a modern way and applied them to the pilot and main tests. In addition, this program is a theme selection activity of the free semester, thus measuring together “career aspirations in technology” for the effectiveness of career exploration, one of the original purposes of the free semester.

#### Artificial intelligence competency

This study used the AI competencies instrument by mixing two test tools related to AI. The first is an attitude test tool for AI for middle school students developed by Kim and Lee (2020a). The AI attitude test tool consists of five constructs, each with “social impact of AI,” “interaction with AI,” “communication with AI,” “emotional exchange with AI,” and “characteristics of AI.”

This study used an eight-item test tool with two constructs, “Social impact of AI” (four items) and “Interaction with AI” (four items) as shown in Table 3. The pilot test included three factors, excluding two out of five factors. The pilot test measured the “characteristics of AI” construct, but it was difficult for students to understand and answer accurately due to ambiguous questions; the reliability was less than 0.7. Accordingly, the study measured only two constructs, “social impact of AI” and “interaction with AI,” excluding the “characteristics of AI” construct.

**Table 3:** Artificial intelligence competency

No	Items
1	If technology advances and AI behaves like humans, bad things are likely to happen to humans
2	I think the future society will be dominated by AI
3	If AI had emotions, I would be anxious
4	I'm worried that AI could have a bad effect on children
5	Dealing with AI is very tense
6	Manipulating and using AI in front of other people is stressful
7	It is terrifying to think that AI can judge something
8	If you work in a workplace that uses AI you may feel anxious
9	I can use AI to improve problems gradually
10	It can detect if the AI is not working and fix the problem
11	I can try new things by reusing what others have programmed (AI)
12	By using AI, I can solve large and difficult problems by dividing them into smaller and easier ones

In addition to the two constructs of “AI attitude,” the researcher used the second test tool to measure “AI performance” in solving technology problems. This study used Choi’s (2014) test developed for computational thinking in robot utilization education. Choi’s test tool consists of three constructs, “computational thinking concept,” “computational thinking performance,” and “computational thinking perspective.” In particular, “computational thinking performance” aims to measure computational thinking centered on problem-solving in robot education. Therefore, it was necessary to modify the construct to fit this study to measure the AI competency implemented in technology education. The researcher modified the test tool of “computational thinking performance” and used it in terms of AI performance competency, focusing on technological problem-solving.

### Data Analysis

The number of participants in this study was 23. Since the sample was small (*n* < 30), the researcher performed a normalization test to examine normality according to the central limit theorem (Kish, 1965). However, most constructs showed normality less than 0.05 in the Kolmogorov–Smirnov, and Shapiro–Wilk tests. Therefore, the researcher conducted the Wilcoxon signed-rank nonparametric test.

## Results

### Program development

**Fig. 1:**
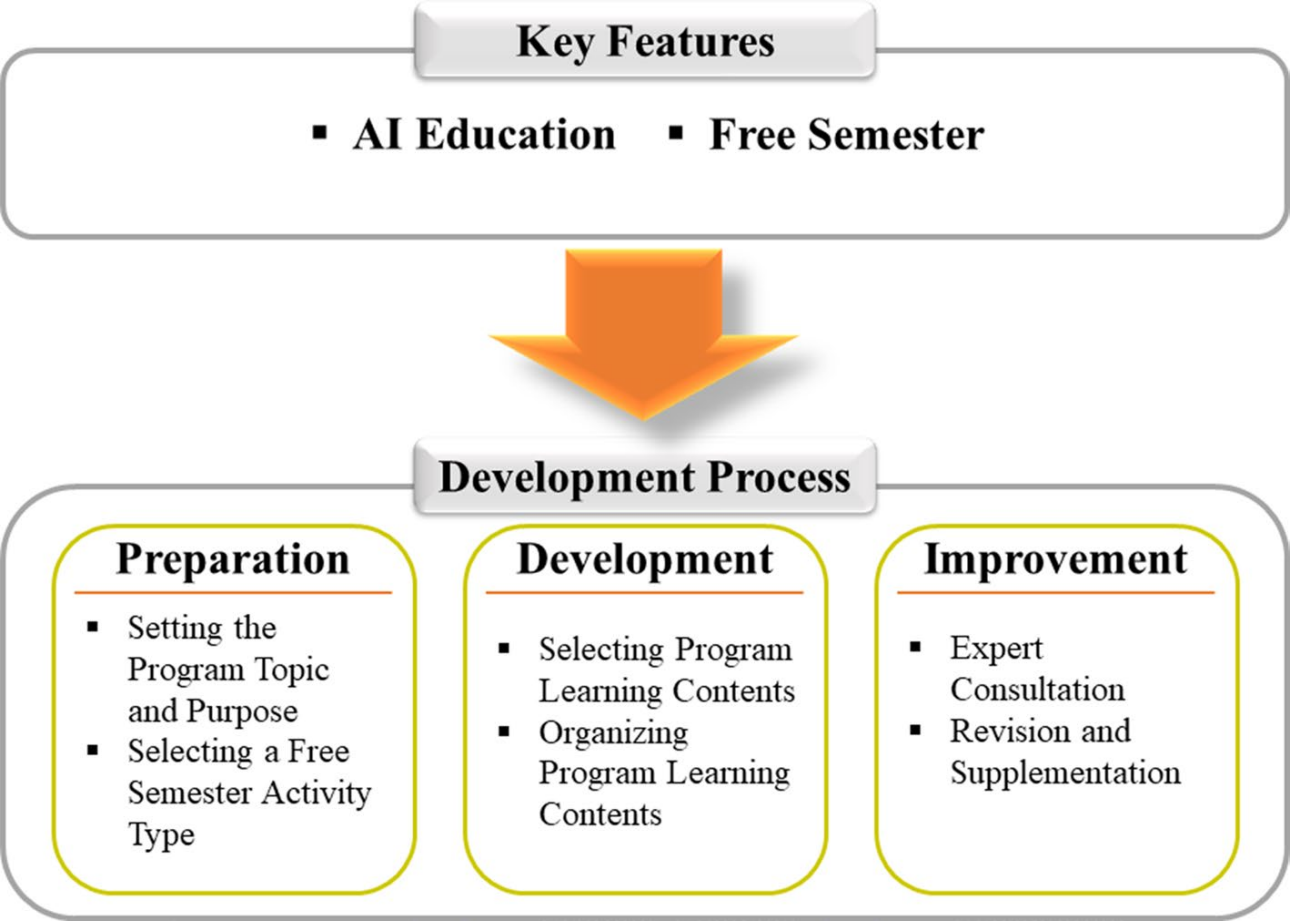
Development Process for Free Semester Program

To develop the middle school AI education theme selection activity for technology education in the free semester, the study used the three steps of “preparation, development, and improvement” suggested by Mager and Beach (1967). The researcher modified and applied each step according to the purpose of the study. The figure below shows the steps of development (See Fig. 1).

In the preparation step, the researcher set the topic and purpose of the AI program based on the literature review. After that, the researcher selected the most suitable program type for study from among the four free semester models, “theme selection activity,” “art-physical activity,” “career exploration activity,” and “club activity” (Ministry of Education, 2015a). In addition, the study did not select learners separately since the free semester is currently implemented only for the first year of middle school.

In the development step, the study extracted AI learning components based on the achievement standards of the practical (technology and home-economics) curriculum. After that, the researcher subdivided the goal of the theme selection activity by each class, selecting and organizing the program contents according to student and teacher roles.

Experts reviewed the program at the improvement stage, and the researcher revised and supplemented the program according to the expert review.

### Preparation

#### Setting the program topic and purpose

As a technology, the trend of patent applications for the latest AI technology is increasing in all fields of industry (Lim et al., 2019). Therefore, considering AI as applied technology, it is not appropriate to confine the topic of an AI education program to a specific unit of technology education.

Based on the literature review, it would be appropriate to develop an AI program in the form of a problem-solving program integrated with a subject (Kim & Han, 2020). For instance, information subject, which is actively researching AI, is approaching the data to realize the purpose of the curriculum. It is significant for technology practitioners to analyze the curriculum and goals of technology education in developing educational program in a technology subject. If one applies AI in technology problem-solving activities, various approaches and convergence will be possible in the technology curriculum.

In a study on the introduction of AI in technology education, Lee and Lee (2021) conducted AI basic and experiential education as an AI free semester program in technology education. In their study, educators implemented a problem-based cooperative learning class format in just a few sections. In another study, students learned the basics and principles of AI through practice with an AI program in technology education, centering on the fourth industrial revolution (Lee et al., 2019). Although it does not directly deal with the AI components, the researcher was able to refer to the achievement standards related to information and communication technology in the 2015 practical arts (technology and home economics) curriculum. Similar to other studies, students learn about the basics, history, and social impact of information and communication technology and experience problem-solving activities (Ministry of Education, 2015b).

In this study, students learned the basics, history, and social impact of AI in the same context and suggested problem-solving activities. This study emphasized the central role of problem-solving, thus excluding excessive professional or vocational education to preserve the uniqueness of technology education. Additionally, per the purpose of the free semester, it should not be a theory-centered or a practice-centered. Therefore, the program aims to cultivate technological literacy to realize the purpose of technology in general subjects.

#### Selecting a free semester activity type

The free semester consists of four activities: theme selection, art–physical, career exploration, and club. The researcher reviewed all four types to effectively apply the AI program. As discussed above, including the literature review, only theme selection activity and art–physical activity types remain in the practical application of AI education in the technology education curriculum. The theme selection activity aims to provide a systematic and professional program tailored to students’ interests. The examples provided by the Ministry of Education (2015a) also include elements related to the fourth industrial revolution; therefore, they are appropriate. Art–physical activity occurs mainly in the arts and physical subjects, and other subjects must include convergence elements. The media sometimes shows examples of applying AI to works of art. However, as this is an AI application field, there are not many cases, and the difficulty level is not appropriate because it requires a high level of programming.

Therefore, considering the organization and operation of the free semester system, theme selection activity is the most appropriate to apply in AI education. Through theme selection activity, teachers provide a professional AI program, and students have opportunities to explore their career paths through various experiences. Thus, this study developed a program in the form of a theme selection activity as a free semester activity.

### Development

#### Selecting program learning contents

After setting the program’s purpose, the researcher determined the learning content for each class. Finally, the researcher extracted content elements related to AI education and organized them from the 2015 revised curriculum (technology and home economics) as shown in Table 4

**Table 4:** AI-related Content and Achievement Standards in Korean National Curriculum

Area	Core concept	Achievement standards and content elements	Al-related content elements
Technology systems	Creation	Explain the characteristics and development process of manufacturing technology, characteristics, and use of materials, and predict the development prospect of manufacturing technology Understand problems related to manufacturingtechnology, creatively explore, realize, and evaluate solutions	4th Industrial Revolution System Design and Automation (Physical Computing)
	Commnication	Understand the detailed elements of each stage of theinformation technology system and explain the information communication process in detail Understand the characteristics and development process of information and communication technologyand explain the characteristics of modern information and communication technology Understand and utilize various types and characteristicsof communication media. Understand problems related to information andcommunication technology, and creatively explore, realize, and evaluate solutions	Data and Information Machine Learning AI Ethics Communication Technology
Technology utilization	Adaptation	Understand changes in society, family, and occupation according to the development of technology, and predict future technology utilization and social changes	Definition of AI AI and Social Change
	Innovations	Find problems in life, come up with ideas, and solve innovation them creatively by using divergent and convergent thinking techniques	Solving Problems Using AI

This study could extract elements related to AI directly or indirectly across a wide area of technology education. For example, in the technology system area, manufacturing technology includes content elements associated with AI such as “the fourth industrial revolution, system design and automation (including physical computing).” Physical computing, automation, etc., are not directly related to AI but can apply AI indirectly as a system to solve problems. Physical activities in the real world can motivate learners to explore and manufacture these systems.

Using computers and AI can link information and communication technology in technology systems to “communication technology problem-solving activities.” In problem-solving activities, learners can experience machine learning. For example, a learner can recognize and learn an image or voice using sensors such as cameras or microphones. In addition, students can learn about data and information and ethical issues of AI with creative activities.

In technology utilization, students can recognize the social impact of AI as a future technology along with occupations. Additionally, in connection with the technology system area, it is possible to find problems in that can be solved by the use of AI.

#### Organizing program learning contents

**Fig. 2:**
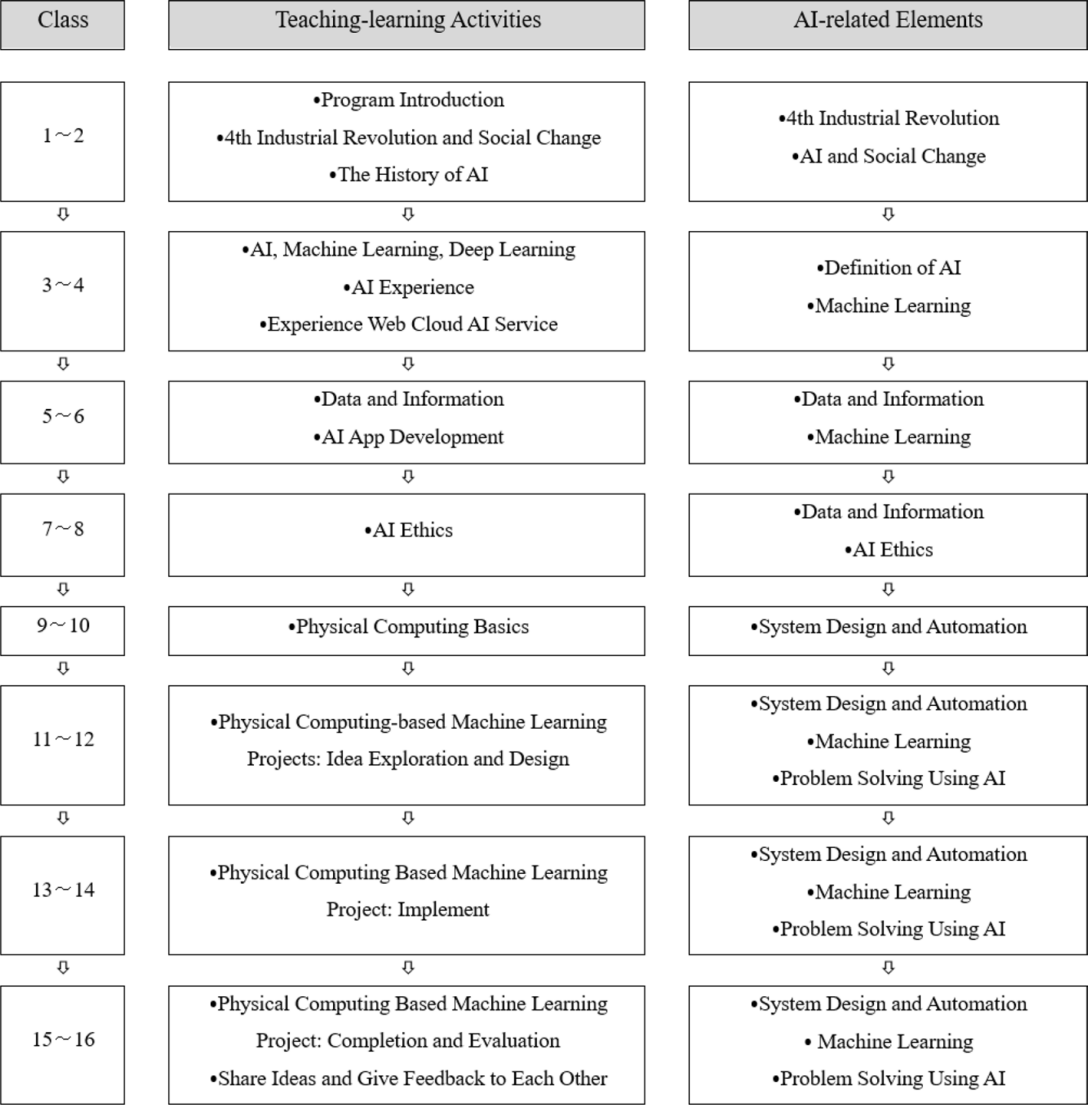
The Developed Program (First Draft)

After arranging the learning contents based on the program’s purpose, the researcher organized the learning content for the 16 h of instruction (See Fig. 2), structured similarly to general technology education courses based on previous studies (Lee & Lee, 2021; Lee et al., 2019; Ministry of Education, 2015b).

### Improvement

#### Expert consultation

In order to review the applicability and validity of the program developed in this study, the researcher asked for consulting from four teachers (educators) and one AI expert with expertise in technology education and convergence education. Table 5 shows the contents of the expert consultation.

**Table 5:** Expert Consultation

Expert	Area of expert	Consulting content
A teacher	More than 10 years of technology and education experience Doctor of curriculuam studies	In accordance with the purpose of the free semester,class for career exploration related to technology include
B teacher	More than 15 years of technology and education experience Master’s in educational psychology Seoul metropolitan office of education supervisior	The program looks very interesting, and the difficulty level seems a bit high
C teacher	More than 15 years of technology and education experience Representative of technology educational professional learing community	I think time for the project is insufficient. There are element of block coding in 3’ ~ 4’ and 5’ ~ 6’ I recommended increasing class of project learning through method such removing duplicate element
D teacher	More than 15 years of technology and education experience Doctor of technology educations	There is a lack of content element based on technology subjects.Is not characterized as a technology problem-sloving activity Difficult level is considered apporopriate
E AI expert	Former s electronics company engoineer Deep learning autonomous driving expert	At the student level, I think the program covers the whole of AI Well

#### Revision and supplementation

**Fig. 3:**
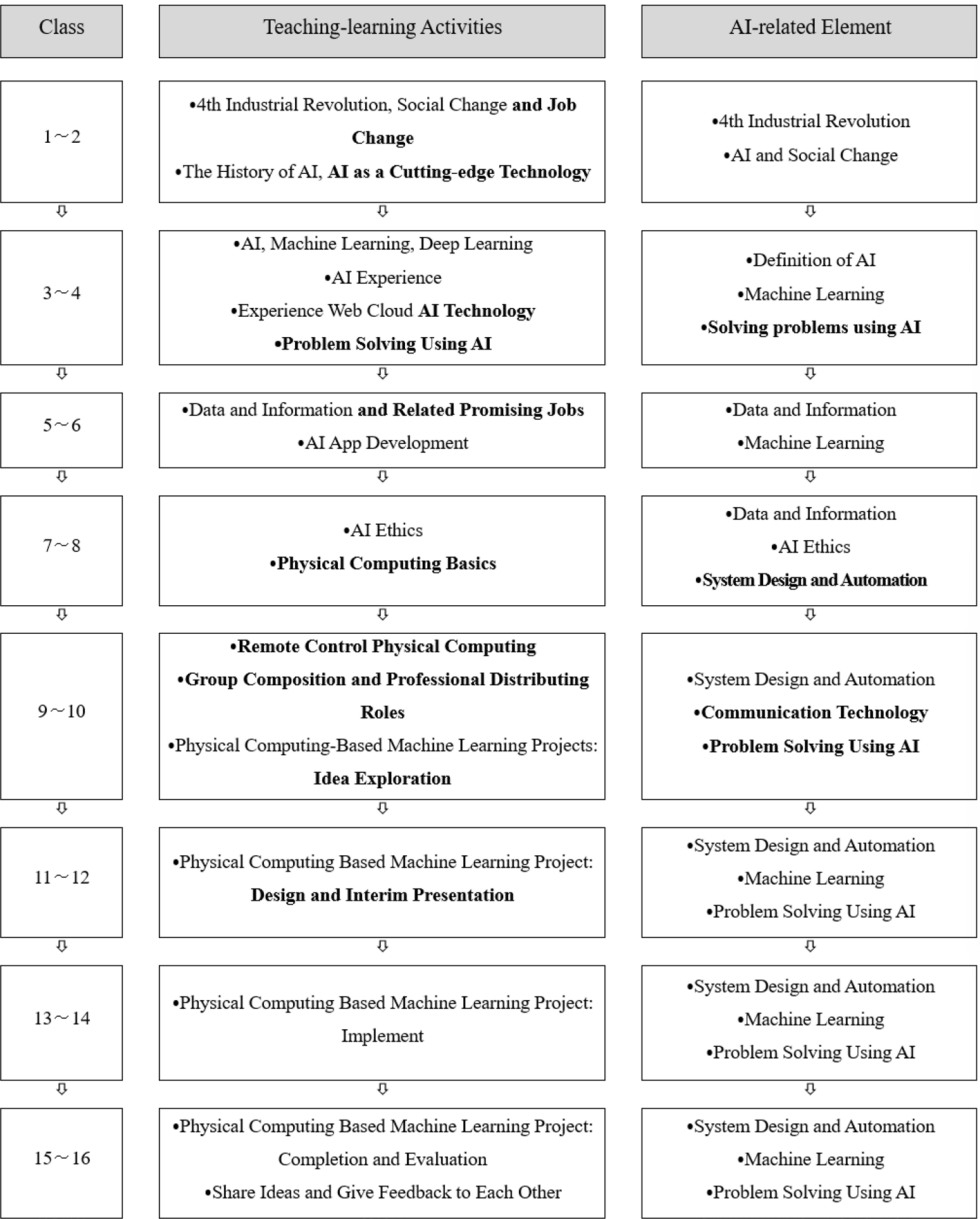
The Final Developed Program

The researcher finally completed the program by modifying and supplementing the program based on the expert consultations. Figure 3 shows the completed program (See Fig. 3).

In the first and second classes, the researcher added the activity of investigating occupations and social changes in the fourth industrial revolution. In addition, the program explained the origins of AI, emphasizing the technological aspect by introducing AI as the latest technology.

Recently, the field of education mainly pursued the latest technology and did not try to deal with the impact of the latest technology on society. However, in the first and second classes, since this is a very familiar area in technology education, the emphasis was on preserving the uniqueness of technology education such as the social change affected by technology (Barlex et al., 2020). Especially, we focused on cutting-edge technology. After that, the program intended to cultivate the students’ ability to evaluate the impact of the latest technology themselves objectively.

In the third and fourth classes, the study changed the term “AI service” to “AI technology.” Additionally, the program added a brief technological problem-solving activity using AI, utilizing AI Experiments; AI drawing; AI music; AI machine learning.

**Fig. 4:**
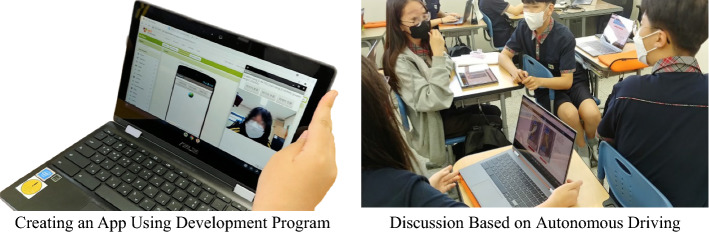
Creating an App and AI ethics Lessons

The program maintained the basic content in the fifth and sixth session classes, introducing data analysts, data engineers, and data scientists as the latest promising jobs (Barlex et al., 2020). In addition, it provided an indirect opportunity to experience the jobs by collecting data and creating an app. Open data was used for data learning, and an app development program was used for app creation. (See Fig. 4)

In the seventh and eighth classes, in AI Ethics, issues such as autonomous driving, AI bias, and privacy were addressed and discussed. Physical computing dealt with Micro:bit coded by educational programming language (EPL) like Scratch block coding.

**Fig. 5:**
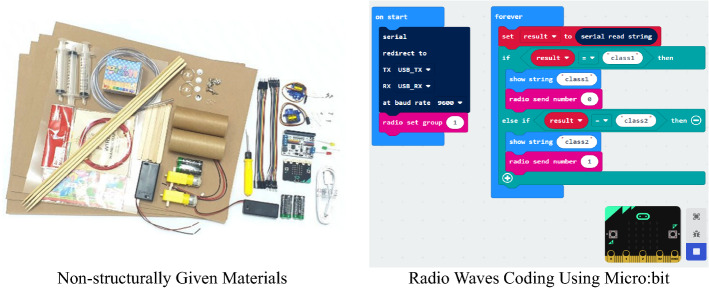
Materials for Project and Coding for Physical Computing

In the ninth to twelfth classes, the students studied remote control technology with Micro:bit (radio communication technology) in physical computing to learn communication technology elements. The students learned how to code the transmitter and receiver using Micro:bit and communicate with friends using radio waves, making it possible to design high-quality projects. In addition, the researcher reorganized the class to enable cooperative learning by adding “group composition and professional distributing roles.” The students were assigned the roles of “team leader,” “engineer,” and “recorder,” thus giving them a more professional experience and a sense of responsibility. Afterward, the students were given an unstructured package, and each team selected a project theme using AI. The program added interim presentations in the eleventh and twelfth classes to share ideas in the results presentation and the process. (See Fig. 5)

**Fig. 6:**
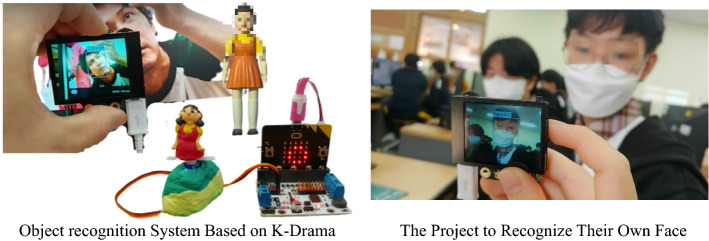
Various Machine Learning Projects

In the thirteenth to sixteenth classes, projects were carried out based on the theme chosen by each team. Various projects have been created, such as the AI that plays an OST when a character is censored, the AI that recognizes friends’ faces, and the AI that represent the famous K-drama. During the final presentation, ideas were shared based on the project portfolio. (See Fig. 6)

### Program implementation and effects

#### Students' attitudinal transition toward technology

The PATT test measured the attitude toward technology in two constructs, “interest in technology” and “career aspirations in technology,” finding a significant increase in both areas (See Table 6). Cronbach’s α of the survey tool was 0.903 in the pretest and 0.886 in the posttest, indicating reliability; “interest in technology” rose from 3.96 to 4.17, and “career aspirations in technology” rose from 3.83 to 4.12. In the Wilcoxon signed-rank test, "interest in technology" and "career aspirations in technology" showed Z values of − 2.047 (*p* < 0.05) and − 2.907 (*p* < 0.01), respectively.

**Table 6:** PATT analysis results

PATT		Pre			Post		N	Z
	M	SD	Mdn	M	SD	Mdn		
Interest in technology	3.96	0.46	3.80	4.17	0.47	3.67	23	− 2.047*
Career aspirations in Technology	3.83	0.64	4.10	4.12	0.47	4.00	23	− 2.907**

^*^p < 0.05, **p < *0.01*

Both constructs showed a significant increase, with a relatively high increase in the “career aspirations in technology” construct. As for the study’s purpose, it was possible to see an increase in the “attitude towards technology” through the AI program. In particular, “interest in technology” and “recognition of job aspects:” rose simultaneously. These findings showed that the developed free semester theme selection activity matched the original purpose of the free semester system.

#### Students' transition toward AI competency

For AI competencies, the study measured three constructs, “social impact of AI,” “interaction with AI,” and “AI performance.” Cronbach’s α of the survey tool was 0.850 in the pretest and 0.866 in the posttest, demonstrating reliability.  Table 7  shows that the average score significantly increased in two of the three areas through the tests before and after the program: “social impact of AI” improved from 3.43 to 4.09, “interaction with AI” improved from 3.74 to 4.00, and “AI Performance” improved from 2.96 to 4.04. In the Wilcoxon signed-ranking test, “social impact of AI” and “AI performance” generated *Z* values of− 3.763 (*p* < 0.001) and − 4.023 (*p* < 0.001), respectively, allowing meaningful observation. On the other hand, “interaction with AI” was − 1.196 (*p* ≧ 0.05), with a higher p-value than 0.05, which was not statistically significant as it had a high probability of significance.

**Table 7:** AI competency analysis results

AI competency		Pre			Post		N	Z
	M	SD	Mdn	M	SD	Mdn		
Social Impact of AI	3.43	0.97	3.50	4.09	0.71	4.25	23	− 3.763***
Interaction with AI	3.74	0.71	3.75	4.00	0.75	4.00	23	− 1.196
AI Performance	2.96	0.65	3.00	4.04	0.71	4.00	23	− 4.023 ***

^***^p < *0.001*

The analysis made it possible to observe that the AI competencies improved after applying the program. In particular, AI performance showed the largest increase. It was possible to confirm that the program developed in this study improved the AI competencies after 16 h of instruction. Simultaneously, the experiment confirmed its value as an AI program.

## Discussion

Because the current technology education lacks research on free semesters and AI education, this study developed an AI program for implementing free semester technology education in schools and analyzed its effectiveness. Technology in-service teachers have difficulty teaching free semester classes (Kim, 2017). In addition, although educational research on AI is active, it is relatively rare in technology education (Kim, 2021).

In this study, starting from an awareness of the above problem, the research purpose was to develop an AI program for implementing in technology education. This study’s program development applied Mager and Beach’s (1967) three steps of “preparation, development, and improvement.” In the preparation step, based on prior research on AI-related technology education, the purpose was to learn AI in general, focusing on problem-solving activities. In particular, the intent was to include technological problem-solving and technological elements to maintain differentiation from other subjects. The researcher analyzed the technology education curriculum in the development step and extracted and organized AI-related elements. In addition, the researcher developed an AI program for learning over 16 h of instruction.

The first class begins by dealing with the impact of the latest technology on society from various perspectives. Recently, the field of education mainly pursued the latest technology and did not try to deal with the impact of the latest technology on society. On the other hand, this is a very familiar area in technology education, and it can preserve the uniqueness of technology education. In addition to the learning topics of general machine learning programs, the program includes system design and automation (physical computing) and communication technology. Although physical computing and communication technology are not directly related to AI, students could use them as tools to solve problems during technological problem-solving activities. On the other hand, the study attempted to expand the information and communication ethics in the previous information and communication technology by adding AI ethics. Although the emphasis on information and communication ethics was not high in domestic technology education, the researchers recognize and emphasize its importance as a new ethical issue in the AI era.

The biggest difference between this AI program and other AI programs is the active sharing-based technology problem-solving activities. The students worked on technological problem-solving as a group activity, and the teacher emphasized cooperation inside and outside the group. Learners were able to share each other’s problem-solving ideas (including source code) from time to time through the web platform. In addition to the teacher’s assessment of the finished product, it was possible to give feedback on ideas sharing activities among students through interim and final presentations. This program is different because most of the previous research on AI in technology education focused on individual activities, conducting cooperative learning only in a few sessions, and previous studies had no presentation (Lee et al., 2019, 2021; Qu et al., 2020).

As a result of data collection and analysis after the program’s application, it was possible to observe the average change in all measured constructs. The observations show the effectiveness of this AI program both in AI education and technology education at the same time. First, “interest in technology” has increased, and in particular, the “career aspirations in technology” construct has changed highly. As a theme selection activity of the free semester, career exploration is an essential element for inclusion. The change in “career aspirations in technology” shows the value of this program as a free semester program. Second, in the AI competency test, the researcher observed an increase in the “social impact of AI, AI performance.” In particular, AI performance shows the largest increase. The AI performance items consist of four questions related to problem-solving, including “problem identification, repair, problem-solving, and problem-solving thinking.” Lim (2020) showed similarities between AI problem-solving processes and technological problem-solving processes. This program showed the effectiveness as a technology education of an AI program developed with a focus on technological problem-solving by applying a similar problem-solving process. In addition, it shows the possibility and validity of technology education in AI education.

This study’s greatest significance is in bringing AI into technology education. As a result of the study, it was possible to confirm the value of the AI free semester program for implementation in technology education as a research purpose. Furthermore, as in the study of Lim (2020), it was possible to confirm the validity of AI education in technology education in the age of AI. In addition, as suggested by Lim (2020), it was possible to confirm the suitability of the AI problem-solving approach integrated with the center of technology education. Similarly, the study could confirm meaningful results of other AI program studies in technology education studies. However, the study’s small sample size and short duration are limitations. Future research could focus on a more in-depth and long-term study with more participants to confirm learners’ change.

## Conclusion and recommendations

Under the “Middle School Free Semester Implementation Plan,” the free semester started nationwide in 2016. From 2020, the free semester expanded to the free year system (Ministry of Education, 2015a). The free semester is a flexible curriculum that improves student-participatory classes so that students can discover their dreams and talents, enabling a variety of career-related experiential activities. However, because the in-service teachers need to spend more time on research and have an increased workload due to the new teaching and evaluation methods, they avoid taking charge of the free semester (Shin et al., 2015).

Meanwhile, the government (2019) designated the field of AI as a national-led project and implemented a reinforcement policy. As a result, interest in AI education is also growing rapidly. After establishing mid- to long-term development plans in each education office, schools are implementing various AI education-related projects (Busan Metropolitan City Office of Education, 2021; Seoul Metropolitan Office of Education, 2021). However, on the other hand, the actual AI education cases have little difference in content from the previous software education, only adding data education (Seoul Metropolitan Office of Education, 2020). In addition, there is a lack of definition and distinction between “education on AI itself” and “education using AI,” adding to the confusion.

This study, starting from the awareness of the above problem, tried to bring AI into technology education by creating an AI education program for immediate implementation. For this, it was necessary to develop an AI program and confirm the value in technology education, confirming the effect of AI. Therefore, the researcher first developed a free semester program in technology education. Second, after implementing the program, the study checked the attitude toward technology and the effectiveness of AI competencies.

## Conclusion

This study developed an AI program for theme selection activity in the free semester for implementation in technology education. After implementing the program, the study’s results led to the following conclusions.

First, the AI program developed as a free semester theme selection activity is effective for career exploration, which is one of the main purposes of the free semester. In the program, learners can explore social changes in the future society, focusing on AI and learning about promising jobs. Students can learn various latest technologies and job changes related to the fourth industrial revolution and experience data-related jobs (data analyst, data scientist, data engineer, and app developer). In addition, as technology developers, students can professionally divide their roles and experience self-directed cooperative development projects. Students can experience various career paths within technology education and positively change attitudes toward technology-related jobs.

Second, this AI program, developed with a focus on technology education, improves AI competencies. The research extracted AI elements dealing with technology education and developed an AI program through expert consultation based on previous research. In the AI program, students learn AI basics, the social impact of AI, data relating to AI, AI ethics, and AI-based problem-solving. In particular, learners can do overall AI elements and cultivate AI competency through technological problem-solving-based projects. Through this program, students can improve their AI competencies, such as their attitude toward AI and AI performance.

Third, the free semester theme selection AI program has value as technology education. AI programs improve AI competencies and attitudes toward technology. In addition, it provides opportunities for career exploration to realize the purpose of the free semester. Thus, the developed free semester theme selection AI program is effective in technology education. Therefore, this program is a free semester AI program should be implemented in technology education.

## Recommendations

The following recommendations are based on the results of this study.

First, further research should include more participants to improve the study’s generalizability. The current study used nonparametric statistical analysis because there were only 23 participants. Thus, future studies should supplement the limitations of statistical methods, including a longer-term study for a comprehensive understanding of the learning results.

Second, there is a need for curriculum research that can improve the perception of technology. The increase in students’ interest in technology in this study suggests a need to change technology classes. Lee (2015) pointed out the difference in perception between technology and technology subjects and the need for various and interesting teaching–learning methods and curriculum research in technology education. Ultimately, it is necessary to study the technology curriculum itself to solve the fundamental problem. Currently, the curriculum does not contain content related to the latest technologies such as AI, metaverse, and autonomous driving. As a result, it is difficult for students to feel interested in the curriculum, and there is a limit to how much improvement can be achieved by teacher teaching–learning methods.

Third, it is necessary to study the latest technology education. Specifically, AI is a very influential field. Furthermore, when excluding research related to AI in elementary education, there were relatively few studies in secondary technology education compared to other subjects. Studies related to the free semester were also lacking. Therefore, educators should treat AI as a technology in the technology curriculum. However, there are only relevant elements, and there is no achievement standard directly related to AI in the current curriculum. Therefore, studying the curriculum aspect of the latest technologies such as AI is necessary. In addition, it is essential to conduct research on technology curriculum to carefully examine changes in the latest technologies like AI and reflect them in the curriculum. Thus, this work should not indiscriminately bring in AI technology. Instead, it is necessary to study AI education in technology education by distinguishing it from science and information subjects, focusing on the purpose of technology subjects.

## Data Availability

All data generated or analyzed during this study are included in this full manuscript.
